# The Relationship Between Nurses' Ethical Behaviours for Protecting Patients' Rights and Patient‐Centred Care Competencies: A Cross‐Sectional Study

**DOI:** 10.1002/nop2.70744

**Published:** 2026-08-13

**Authors:** Melek Aksut Ozturk, Beratiye Oner

**Affiliations:** ^1^ Ordu Akkus Public Hospital Ordu Turkiye; ^2^ Department of Nursing, Faculty of Health Sciences Lokman Hekim University Ankara Turkiye

**Keywords:** competency, ethics, nursing, patient rights, patient‐centred care

## Abstract

**Aim:**

To determine the association between nurses' ethical behaviours for protecting patients' rights and their competencies in delivering patient‐centred care.

**Design:**

A cross‐sectional, descriptive, and correlational design was used.

**Methods:**

This study involved 397 nurses from diverse healthcare institutions across Turkiye (December 2024–February 2025). Data were collected using the “Nurses' Ethical Behaviours for Protecting Patients' Rights Scale” and the “Patient‐Centred Care Competency Scale”. Due to multicollinearity and the bounded outcome variable, each ethical behaviour subscale was evaluated in a separate logistic regression model controlling for demographic confounders, with patient‐centred care competency dichotomized as high versus less‐than‐high. Spearman rank‐order correlation coefficients, Mann–Whitney U tests, and Kruskal‐Wallis tests were also used.

**Data Sources:**

Nurses in public, university, and foundation hospitals across Turkiye.

**Results:**

Most participants were female with a median age of 29 years. Nurses who had received patient rights education demonstrated significantly higher patient‐centred care competency scores. Strong positive correlations were observed between all ethical behaviour subscales and patient‐centred care dimensions. All five ethical behaviour dimensions were independently associated with a reduced likelihood of less‐than‐high patient‐centred care competency, with the strongest associations for patient values and choices and benefit‐not‐harming.

**Conclusion:**

All dimensions of nurses' ethical behaviour in protecting patients' rights are independently associated with patient‐centred care competency. Integrating ethics and patient rights education into nursing curricula and continuing professional development is essential, with emphasis on experiential approaches. Adequate staffing and institutional support are structural prerequisites for translating ethical values into consistent patient‐centred care delivery.

**Implications for the Profession and/or Patient Care:**

This study supports integrating ethics and patient rights into nursing education and professional development. Adequate staffing and institutional support are structural prerequisites for consistent care delivery, with implications for healthcare policy and workforce planning.

**Impact:**

*What problem did the study address?* The study addressed the lack of empirical data linking nurses' ethical behaviors related to patient rights protection with their patient‐centred care competencies and identified methodological limitations in prior analyses through multicollinearity diagnostics. *What were the main findings?* All five ethical behavior dimensions were significantly associated with patient‐centred care competency in separate logistic regression models. Strong positive correlations were observed between ethical behavior and patient‐centred care dimensions. Nurses who received patient rights education demonstrated significantly higher patient‐centred care competency scores. *Where and on whom will the research have an impact?* The findings will impact nursing educators, healthcare administrators, and frontline nurses by informing curriculum reforms, institutional training programs, and ethical practice standards, thereby enhancing patient‐centred care delivery across healthcare settings.

**Reporting Method:**

This study employed a cross‐sectional, descriptive, and correlational design using validated self‐report scales. Data were collected via an online survey from 397 nurses providing inpatient and outpatient care in public, university, and foundation hospitals across Turkiye. Following multicollinearity diagnostics, statistical analyses included Spearman's rank‐order correlations and separate logistic regression models for each ethical behavior subscale, with patient‐centred care competency operationalized as a binary outcome (high vs. less‐than‐high competency).

**Patient or Public Contribution:**

No direct patient or public involvement occurred in the study design, data collection, or analysis stages. However, the study indirectly reflects patient‐centred values by examining how nurses' ethical behavior is associated with care quality from the patient's perspective.

## 1 Introduction

Nurses occupy a uniquely central position in healthcare delivery, assuming direct and continuous responsibility for patient welfare across all care settings. This proximity places ethical conduct at the core of nursing practice, where decisions involving patient autonomy, confidentiality, informed consent, and the protection of patient rights are encountered daily rather than exceptionally (Varkey 2021). Empirical evidence confirms that nurses' adherence to ethical principles remains a defining feature of professional practice, shaping both the quality of care delivered and the trust patients place in healthcare institutions (Wong et al. 2025). The formalization of nursing ethics, initiated with the American Nurses Association's Code of Ethics and subsequently developed through the International Council of Nurses, reflects the profession's enduring commitment to these responsibilities (American Nurses Association 2015; International Council of Nurses 2021). Within this ethical framework, the protection of patient rights has emerged as a particularly critical domain, encompassing nurses' active advocacy for patients' informational, decisional, and privacy rights in increasingly complex institutional environments. In contemporary healthcare environments, nurses' ethical responsibilities extend beyond the bedside to include digital professionalism, privacy in online communication, and the ethical use of health technologies, dimensions that further underscore the breadth and complexity of nursing ethics in the twenty‐first century (Wang et al. 2019).

Patient‐centred care (PCC) is defined as care that respects and responds to individual patient preferences, needs, and values while actively promoting patient participation in decision‐making (Marchand et al. 2020). First formally recognized in the Institute of Medicine's 2001 Report (Institute of Medicine 2001) “Crossing the Quality Chasm”, PCC has since emerged as a transformative paradigm in healthcare quality. Nurses are central to PCC delivery precisely because their ethical responsibilities, respect for autonomy, nonmaleficence, and privacy protection mirror the foundational dimensions of person‐centred practice. Research demonstrates that PCC improves patient satisfaction, increases active participation in treatment, and reduces healthcare conflicts (Holden et al. 2020), and is associated with improved patient outcomes and enhanced organizational performance (Rathert et al. 2013; Kwame and Petrucka 2021). This conceptual alignment between nursing ethics and PCC suggests that nurses' ethical sensitivity in protecting patient rights may constitute a meaningful determinant of their PCC competency. However, empirical evidence directly linking specific ethical behaviour dimensions to PCC competency remains limited, particularly in the Turkish nursing context. The present study therefore aimed to examine this relationship and identify which dimensions of ethical behaviour are most strongly associated with PCC competency.

## 2 Background

Ethical principles in healthcare have evolved over centuries through the dynamic interplay of moral philosophy, religious beliefs, cultural traditions, and legal frameworks (Orhan 2007), providing a robust structure for regulating professional behaviours and upholding moral integrity within organizational contexts (Utlu 2016). The concept of protecting patient rights is closely intertwined with nurses' ethical responsibilities (Wong et al. 2025). Empirical evidence consistently demonstrates that ethical nursing conduct has measurable consequences for patient‐related outcomes (Rathert et al. 2013; Ulrich et al. 2010). When nurses actively protect patient rights through informed consent facilitation, privacy safeguarding, and support for autonomous decision‐making, patients report higher levels of satisfaction, trust, and therapeutic engagement (Rathert et al. 2013; Kwame and Petrucka 2021). Conversely, ethical violations or lapses in protecting patient rights have been associated with reduced patient safety, diminished trust in healthcare providers, and poorer care experiences (Ulrich et al. 2010). Studies have also shown that nurses working in environments with weak ethical climates are more likely to experience moral distress, which, in turn, compromises their capacity for advocacy and individualized care delivery (Ulrich et al. 2010). These findings collectively underscore that ethical behaviour in nursing is not merely a professional obligation but a direct determinant of care quality and patient outcomes.

Beyond individual patient interactions, the integration of patient rights protection into nursing practice is thus a critical component of quality care delivery and sustainability (Utlu et al. 2016). Despite the recognized importance of both ethical nursing practice and PCC, the intersection between these domains remains underexplored in empirical literature. Ethical principles such as respect for autonomy, beneficence, and non‐maleficence not only form the foundational ethical framework of healthcare but also serve as operational mechanisms that enable PCC in clinical settings. For instance, respecting patient autonomy aligns with PCC's emphasis on shared decision‐making, while beneficence and non‐maleficence relate directly to tailoring care to individual needs and minimizing harm (Scholl et al. 2014). According to Entwistle and Watt 2013, person‐centred care inherently embodies ethical principles by recognizing patients as moral agents and promoting their capabilities to participate in care processes. This ethical lens positions PCC not merely as a communication or service model, but as a moral imperative grounded in justice, dignity, and empowerment. However, much of the literature has treated ethics and PCC as distinct constructs, with limited integration in empirical nursing research. Recent reviews highlight that while ethical dilemmas have intensified during the COVID‐19 pandemic, empirical evidence linking these ethical experiences to measurable PCC competencies remains scarce (Aydogdu 2022; Oh and Gastmans 2023). Most studies have examined moral distress or ethical sensitivity in isolation, without examining how such ethical behaviours, particularly those that protect patient rights, are operationalized in the delivery of person‐centred care. Emerging evidence suggests that ethical sensitivity enhances nurses' ability to detect patients' psychosocial, emotional, and existential needs, fostering empathetic communication, trust, and mutual respect, core dimensions of PCC (Milliken 2018). Moreover, a favourable ethical climate, characterized by open dialogue, team support, and ethical leadership, has been associated with reduced moral distress and enhanced patient advocacy, both of which contribute to high‐quality person‐centred care (Schluter et al. 2008; Donkers et al. 2021).

**FIGURE 1:**
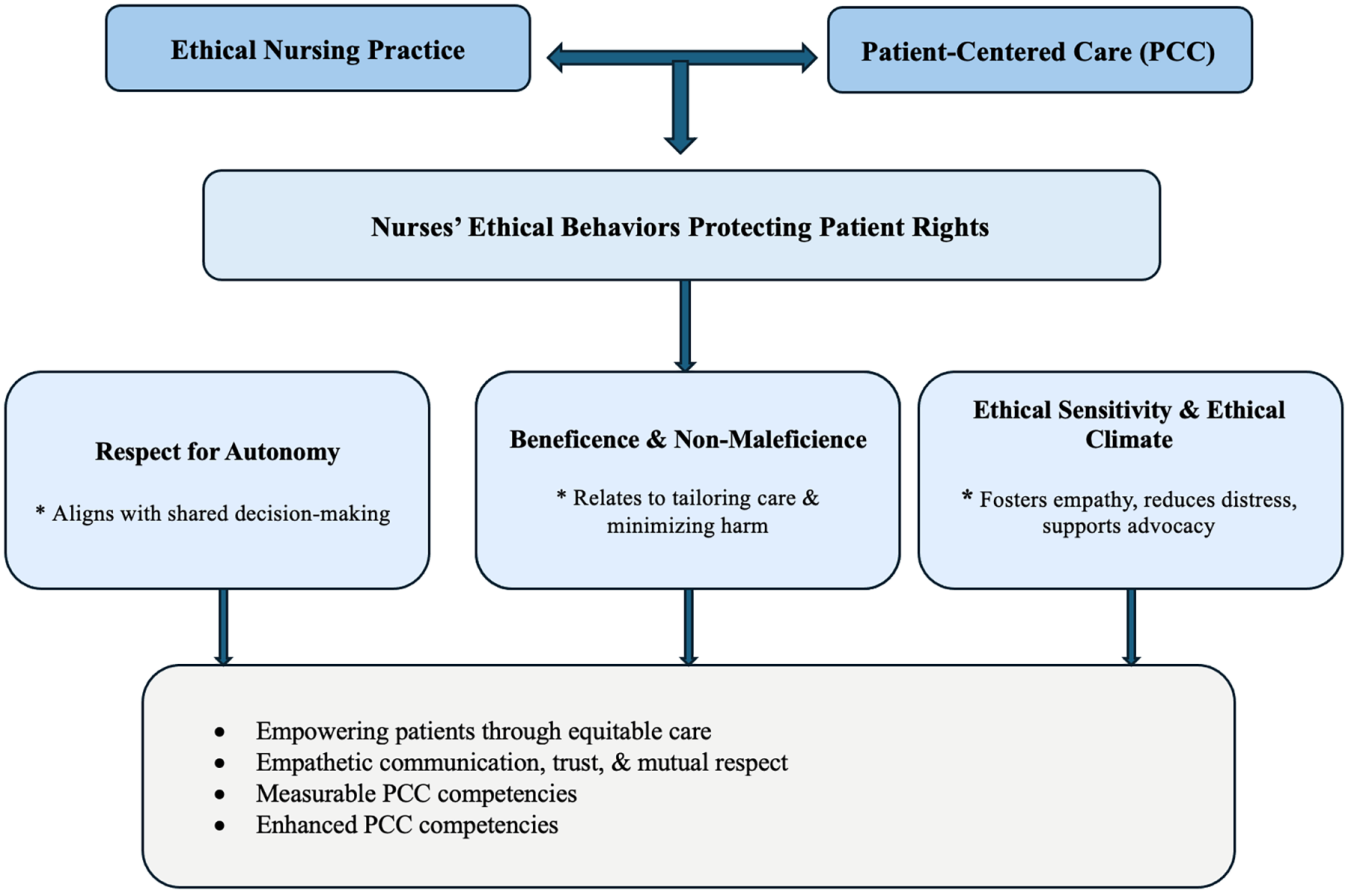
Conceptual framework of the study. PCC, patient‐centred care.

Drawing from this body of literature, the present study is grounded in a conceptual framework that positions ethical nursing behaviour, particularly actions that protect patient rights, as a key enabler of PCC competencies. As illustrated in Figure 1, ethical principles (autonomy, beneficence, non‐maleficence) inform ethical sensitivity and climate, which shape nurses' ethical behaviours in practice. These behaviours, in turn, foster competencies such as shared decision‐making, individualized care, empathy, and respect, hallmarks of PCC. This framework also considers moderating factors such as nurses' demographics and professional experience, which may influence the strength of these relationships.

This framework was developed based on integrative models of Scholl et al. (2014), Entwistle and Watt (2013), Milliken (2018), Schluter et al. (2008), and Donkers et al. (2021). It conceptualizes nurses' ethical behaviours in protecting patient rights as mechanisms that enhance PCC competencies through ethical sensitivity, empathy, communication, and empowerment processes. The conceptual framework (Figure 1) explicitly illustrates how ethical nursing principles—autonomy, beneficence, and non‐maleficence—serve as mechanisms that operationalize person‐centred care through ethical sensitivity, empathy, communication, and empowerment. Within this model, nurses' ethical behaviours that protect patients' rights serve as the foundational drivers of PCC competencies such as shared decision‐making, individualized care, and patient advocacy. This integrative framework bridges the conceptual gap between ethical theory and clinical practice, positioning ethics not merely as a philosophical underpinning but as a measurable determinant of patient‐centred outcomes.

## 3 The Study

### 3.1 Aim

This study aimed to determine the levels of Nurses' Ethical Behaviour for Protecting Patients' Rights (NEBPPR) and their PCC competencies, as well as the factors associated with these behaviours, and to examine the association between nurses' ethical behaviours and their PCC competencies.

## 4 Methods

### 4.1 Design

A cross‐sectional, descriptive, and correlational design was employed.

### 4.2 Sample and Setting

Data were collected between December 2024 and February 2025 via an online survey distributed using a snowball sampling strategy. The target population consisted of nurses providing direct patient care in both inpatient and outpatient units at public, university, and foundation hospitals across Turkiye. Both unit types were intentionally chosen because patient‐centred care competency was examined across direct care settings rather than being confined to a single care context. The minimum required sample size was calculated using G*Power 3.1.9.7. For a two‐tailed Spearman correlation analysis, assuming a small effect size (*r* = 0.20), α = 0.05, and power (1–β) = 0.95, the required minimum sample size was *n* = 262 participants. A 20% attrition buffer was applied, yielding a recruitment target of at least 315 participants. Of the 413 surveys completed, 397 met the inclusion criteria and were included in the final analysis, substantially exceeding the minimum requirement. For the logistic regression analyses, the events‐per‐variable criterion was also considered: with 49 participants in the less‐than‐high competency category and a maximum of four variables per model (one NEBPPR subscale and three demographic confounders), the events‐per‐variable were 12.25, which meets the recommended minimum threshold of 10.

### 4.3 Inclusion and Exclusion Criteria

The inclusion criteria for the study were: (a) having at least 1 year of nursing experience, and (b) actively working as a nurse in inpatient or outpatient care units providing direct patient care in Turkiye. The exclusion criteria were: (a) working as a nurse in non‐patient care units, such as education and quality units, (b) holding a nursing managerial position, and (c) incomplete survey responses.

### 4.4 Data Collection

Surveys were administered via Google Forms. Participants provided electronic informed consent before responding.

### 4.5 Instruments With Validity and Reliability

#### 4.5.1 Personal Information Form

It was developed by the researchers based on the literature (Adiguzel et al. 2011; Tan et al. 2015) and consists of 21 questions designed to assess the sociodemographic (age, gender, marital status, etc.) and professional characteristics of the participants, including educational background, years of experience in the profession and institution, unit type, work schedule, weekly working hours, professional satisfaction, ethics, patient rights, and training received on patient care competencies. Specifically, the form included items assessing whether participants had received ethics training, whether they had received patient rights education and its primary source, their familiarity with PCC, their perception of PCC necessity in clinical practice, and the barriers they identified to PCC implementation.

#### 4.5.2 Nurses' Ethical Behaviour for Protecting Patients' Rights (NEBPPR) Scale

This 28‐item, 5‐point Likert scale, developed by Eyuboglu et al. (2022), measures nurses' ethical behaviour in protecting patients' rights across five sub‐dimensions: respect for information and decision‐making rights, providing fair care, providing benefit‐not harming, respect for patient values and choices, and attention to privacy. The score range is from 28 to 140, with higher scores indicating more positive ethical behaviours. In the original scale development study, the Cronbach's Alpha coefficient was reported as 0.840 (Eyuboglu et al. 2022). In the current study, the Cronbach's Alpha coefficient was 0.861 for the total scale, indicating high internal consistency in the present sample.

#### 4.5.3 Patient‐Centred Care (PCC) Competency Scale

PCC Competency Scale, developed by Hwang (2015) and adapted into Turkish by Arslanoglu and Kirilmaz (2019) is a measurement tool consisting of four sub‐dimensions: respecting patients' perspectives, promoting patient involvement in care processes, providing for patient comfort, advocating for patients, and 17 items on a 5‐point Likert scale. Items are scored from 1 (strongly disagree) to 5 (strongly agree), with higher scores indicating higher PCC competencies. In the Turkish adaptation study, the overall Cronbach's Alpha coefficient was reported as 0.850 (Arslanoglu and Kirilmaz 2019). In the current study, the overall Cronbach's Alpha coefficient was 0.941, indicating high internal consistency in the sample.

### 4.6 Data Analysis

Data were analysed using IBM SPSS Statistics (Version 29.0; IBM Corp., Armonk, NY, USA). Statistical significance was set at *p* < 0.05. The Kolmogorov–Smirnov Test indicated a non‐normal distribution of continuous variables (*p* < 0.001), which guided the use of nonparametric methods throughout. Descriptive statistics, including frequencies, percentages, medians, and interquartile ranges (IQR), were used to summarize participant characteristics. The reliability of both scales was evaluated using Cronbach's Alpha coefficient. Spearman's rank‐order correlation coefficients were used to examine associations between NEBPPR subscales and PCC competency dimensions and total scores.

To examine the association between ethical behaviour dimensions and PCC competency, logistic regression analysis was employed rather than linear regression for two reasons. First, multicollinearity diagnostics revealed severely elevated Variance Inflation Factor (VIF) values across NEBPPR subscales when entered simultaneously into a combined model (range: 28.6–125.5), indicating that a single combined regression model was methodologically inadequate. Second, given the bounded nature of the PCC competency outcome (theoretical range: 17–85; observed minimum: 34) and the highly skewed distribution of competency levels (87.7% high competency), PCC competency was dichotomized as high (≥ 63) versus less‐than‐high (≤ 62), and binary logistic regression was applied. Each NEBPPR subscale was therefore evaluated in a separate logistic regression model, with gender, years of experience, and education level included as demographic confounders. Odds ratios (OR) and 95% confidence intervals are reported for each model. Mann–Whitney *U* tests were used to compare continuous PCC competency scores between groups for educational variables, and the Kruskal‐Wallis test was used for multi‐group comparisons.

### 4.7 Ethical Considerations

The research was conducted with the necessary permissions from the relevant Non‐Interventional Clinical Research Ethics Committee (Decision Number: 2024/277‐Code Number: 2024267). In accordance with the Declaration of Helsinki, the study's purpose, the protection of personal data and confidentiality, and the voluntary nature of participation were clearly explained to participants, and online informed consent was obtained. This study adhered to the EQUATOR Network reporting standards and specifically followed the Strengthening the Reporting of Observational Studies in Epidemiology (STROBE) checklist for cross‐sectional research to enhance methodological transparency, reproducibility, and reporting accuracy (Data S1) (von Elm et al. 2007). The completed STROBE checklist, indicating the manuscript location of each reported item, is provided as a Supporting Information (Data S1).

## 5 Results

### 5.1 Demographic and Professional Characteristics

The demographic and professional characteristics of the 397 participants are summarized in Table 1; full distributions are provided in Appendix S1. The sample was predominantly female (67.8%), with a median age of 29 years (IQR: 26–34), ranging from 20 years to 57 years. Most participants held a bachelor's degree (59.7%); given the small number of participants with doctoral qualifications, holders of master's and doctoral degrees were combined into a single postgraduate category for analysis. The majority had 1–5 years of professional experience, with a median of 6 years (IQR:3–12), ranging from 1 year to 36 years. Participants were distributed across diverse hospital types and geographic regions, with the largest proportions working in state hospitals (31.2%) and the Black Sea region (36.3%). Private and private specialty hospitals were combined into a single category due to small cell sizes. The majority (80.4%) worked in an inpatient care setting (Table 1 and Appendix S1).

**TABLE 1:** Demographic, professional, and educational characteristics of participants (*n* = 397).

Variables		*n* (397)	%
Age (years)	Median (IQR): 29 (26–34)		
Gender	Female	269	67.8
	Male	128	32.2
Education	Health Vocational High School Graduate	64	16.1
	Associate degree	33	8.3
	Bachelor's degree	237	59.7
	Graduate degree	63	15.9
Institution type	State hospital	124	31.2
	City hospital	101	25.4
	Private (Foundation) hospital	75	19.0
	Training and research hospital	48	12.1
	University hospital	49	12.3
Years of experience	Median (IQR): 6 (3–12)		
Ethics training status	Yes	312	78.6
	No	85	21.4
Patient rights education status	Yes	346	87.2
	No	51	12.8
Location of patient rights training^a^	Current workplace	197	57.8
	Undergraduate/Graduate education	121	35.5
	Certificate program	23	6.7

Abbreviation: IQR, interquartile range.

^a^ Five participants who indicated receiving patient rights education but did not specify the source were excluded from this analysis (*n* = 341).

### 5.2 Education and Knowledge Status

Details of participants' education and knowledge status are presented in Appendix S2. Most nurses had received ethics training (78.6%) and were familiar with PCC (82.4%). Nearly all (96.2%) considered a PCC approach necessary in clinical practice. Staff shortage was identified as the most frequently reported barrier to PCC implementation (72.5%). The majority (85.9%) had also received patient rights education, primarily through their current workplace (57.8%) (Table 1).

### 5.3 The Relationship Between PCC Competence and Educational Factors

Participants who had received patient rights education demonstrated significantly higher PCC competency scores (Median = 74.0, IQR: 68–83) compared to those who had not (Median = 68.0, IQR: 63–78; U = 11,436, *p* = 0.001). Similarly, nurses who had received ethics training scored significantly higher (Median = 74.0, IQR: 67.5–83) than those who had not (Median = 71.5, IQR: 64–78.8; U = 14,794, *p* = 0.009). These findings are summarized in (Table 2).

**TABLE 2:** Comparison of PCC competency scores by patient rights education and ethics training status.

	*n*	Median (IQR)	U/H	*p*
Patient rights education			*U* = 11,436	0.001
Yes	346	74.0 (68–83)		
No	51	68.0 (63–78)		
Ethics training			*U* = 14,794	0.009
Yes	319	74.0 (67.5–83)		
No	78	71.5 (64–78.8)		
Source of patient rights education			*H* = 2.575	0.276
Undergraduate/postgraduate education	121	75.0 (68–83)		
Workplace training	197	74.0 (68–82)		
Certified training programme	23	71.0 (63.5–80.5)		

*Note:* Mann–Whitney U test used for two‐group comparisons; Kruskal‐Wallis H test used for multi‐group comparison.

Abbreviations: IQR, interquartile range; PCC, patient‐centred care.

Regarding the source of patient rights education, no statistically significant difference in PCC competency scores was observed across education sources, undergraduate/postgraduate education (*n* = 121, Median = 75.0, IQR: 68–83), workplace training (*n* = 197, Median = 74.0, IQR: 68–82), or certified training programs (*n* = 23, Median = 71.0, IQR: 63.5–80.5) (Kruskal‐Wallis H = 2.575, *p* = 0.276), suggesting that the benefit of patient rights education on PCC competency may be independent of the setting in which it was received (Table 2).

### 5.4 Scale Scores of NEBPPR and PCC Competencies

High scores were obtained for all subscales of both instruments. The total score for the NEBPPR Scale was 120.00 (IQR: 111–126, range: 68–138), while the total score for the PCC Competency Scale was 74.00 (IQR: 67–82, range: 34–85) (Table 3). The relatively narrow interquartile ranges indicate homogeneity in the participants' responses on both scales. These results demonstrate that nurses exhibit a high level of ethical behaviour in protecting patients' rights and possess strong PCC competencies, supporting the adoption of ethics‐based and patient‐centred approaches in nursing practice.

**TABLE 3:** Median and interquartile range values of NEBPPR and PCC competency scales (*n* = 397).

	Scale/subscale	Median	Q1–Q3	Min	Max
NEBPPR Scale	Respect for information and decision‐making rights	34	31–38	13	43
	Providing fair care	28	25–29	6	30
	Providing benefit‐not harming	24	21–25	10	25
	Respect for patient values and choices	17	16–19	4	20
	Attention to privacy	19	17–20	8	20
	NEBPPR total	**120**	**111–126**	**68**	**138**
PCC Competency Scale	Respecting patients' perspectives	25	23–30	6	30
	Promoting patient involvement in care processes	21	19–25	9	25
	Providing for patient comfort	14	12–15	6	15
	Advocating for patients	14	12–15	5	15
	PCC total	**74**	**67–82**	**34**	**85**

*Note:* Bold values indicate totals.

Abbreviations: Max, maximum; Min, minimum; NEBPPR, nurses' ethical behaviour for protecting patients' rights; PCC, patient‐centred care; Q1, 25th percentile; Q3, 75th percentile.

### 5.5 The Relationship Between NEBPPR and PCC Competencies

Spearman's rank‐order correlation analysis revealed significant positive relationships between all NEBPPR subscales and PCC Competency subscales and total scores (Table 4). The strongest correlation was observed between NEBPPR Total and PCC Total (ρ = 0.683, *p* < 0.001), followed by Benefit‐Not Harming and PCC Total (ρ = 0.668, *p* < 0.001). The weakest cross‐scale correlation was between Providing Fair Care and Promoting Patient Involvement (ρ = 0.128, *p* = 0.010). All correlations were statistically significant and positive, indicating that higher ethical behaviour scores across all NEBPPR dimensions are consistently associated with higher PCC competency scores.

**TABLE 4:** Spearman correlation matrix between NEBPPR subscales and PCC competency subscales variables.

NEBPPR scale	Respecting perspectives	Promoting involvement	Patient comfort	Advocating for patients	PCC total
PCC competency subscales and total score					
1. Respect for information and decision‐making rights	0.548**	0.539**	0.517**	0.461**	0.596**
2. Providing fair care	0.180**	0.128*	0.243**	0.249**	0.206**
3. Benefit‐not harming	0.634**	0.556**	0.594**	0.583**	0.668**
4. Patient values and choices	0.553**	0.492**	0.462**	0.447**	0.570**
5. Attention to privacy	0.560**	0.521**	0.472**	0.475**	0.584**
NEBPPR total	**0.644** **	**0.583** **	**0.602** **	**0.565** **	**0.683** **

*Note:* Bold values indicate totals.

Abbreviations: NEBPPR, nurses' ethical behaviour for protecting patients' rights; PCC, patient‐centred care.

* *p* < 0.05.

** *p* < 0.001.

### 5.6 Association Between NEBPPR Dimensions and PCC Competency

As outlined in Section 4.6, each NEBPPR subscale was examined using a separate binary logistic regression model to avoid multicollinearity bias, as preliminary diagnostics indicated substantially elevated VIF values when subscales were included in a combined model. Additionally, due to the highly skewed distribution of PCC competency scores and the imbalance between categories (87.7% high competency vs. 12.3% less‐than‐high competency), PCC competency was dichotomized as high (≥ 63) and less‐than‐high (≤ 62). Each model controlled for gender, years of experience, and education level as potential confounders. Among the 397 participants, 348 (87.7%) demonstrated high PCC competency, whereas 49 (12.3%) demonstrated less‐than‐high competency. Results from the five separate logistic regression models are presented in Table 5.

**TABLE 5:** Logistic regression models examining association between NEBPPR subscales and PCC competency (*n* = 397). Modelled event: Less‐than‐high PCC competency (reference category: High PCC competency).

NEBPPR Subscale	B	SE	OR	95% CI	*p*
Respect for Information and Decision‐Making Rights	−0.272	0.038	0.762	0.708–0.821	< 0.001**
Providing Fair Care	−0.068	0.027	0.934	0.886–0.985	0.011*
Benefit‐Not Harming	−0.382	0.06	0.683	0.607–0.768	< 0.001**
Patient Values & Choices	−0.396	0.062	0.673	0.596–0.760	< 0.001**
Attention to Privacy	−0.368	0.062	0.692	0.613–0.782	< 0.001**

*Note:* A full model example (intercept, covariates, and variable of interest) is presented in Appendix S3.

Abbreviations: B, unstandardized logistic regression coefficient; CI, confidence interval; OR, odds ratio; SE, standard error.

* *p* < 0.05.

** *p* < 0.001.

All five NEBPPR dimensions were significantly associated with PCC competency. In all models, higher scores on ethical behaviour subscales were associated with lower odds of less‐than‐high PCC competency, indicating a protective relationship between ethical behaviour and PCC competency (all ORs < 1, all *p*‐values < 0.05). The strongest associations were observed for patient values and choices (OR = 0.673, 95% CI: 0.596–0.760, *p* < 0.001) and benefit‐not harming (OR = 0.683, 95% CI: 0.607–0.768, *p* < 0.001), suggesting that higher scores in these domains were associated with a substantially reduced likelihood of lower competency levels. Attention to privacy (OR = 0.692, 95% CI: 0.613–0.782, *p* < 0.001) and respect for information and decision‐making rights (OR = 0.762, 95% CI: 0.708–0.821, *p* < 0.001) were also strongly and statistically significantly associated. Although still significant, providing fair care showed the weakest association with PCC competency (OR = 0.934, 95% CI: 0.886–0.985, *p* = 0.011), indicating a smaller effect size compared with other subscales.

## 6 Discussion

This study examined the association between nurses' levels of ethical behaviour in protecting patient rights and their PCC competencies. The findings revealed that nurses' sensitivity to ethical principles is significantly associated with PCC practices, and all five NEBPPR dimensions are negatively associated with the likelihood of less‐than‐high PCC competency in separate logistic regression models. These results align with Epstein and Street's (Epstein and Street 2007) framework, demonstrating that effective PCC is fundamentally linked to ethical principles, including information sharing, respect for patient autonomy, privacy protection, and the benefit‐harm balance.

The strongest association was observed for “patient values and choices” (OR = 0.673, *p* < 0.001), followed by “benefit‐not harming” (OR = 0.683, *p* < 0.001) and “attention to privacy” (OR = 0.692, *p* < 0.001), suggesting that nurses' respect for patients' personal values, commitment to nonmaleficence, and attention to privacy are most strongly associated with high PCC competency. These findings are consistent with frameworks positing that respect for patient autonomy, nonmaleficence, and privacy protection constitute foundational mechanisms of person‐centred practice (Scholl et al. 2014; Entwistle and Watt 2013). “Respect for information and decision‐making rights” also demonstrated a significant association (OR = 0.762, *p* < 0.001), supporting the Institute of Medicine's (Institute of Medicine 2001) emphasis on patients assuming active decision‐making roles in care processes. “Providing fair care” showed the weakest, though still significant, association (OR = 0.934, *p* = 0.011), suggesting that equitable treatment, while ethically essential, may be less directly linked to PCC competency than other ethical behaviour dimensions in this sample. However, not all evidence uniformly supports this relationship. Several studies have reported that structural constraints, including understaffing, high patient‐to‐nurse ratios, and limited institutional support, may override individual ethical orientation as determinants of PCC quality, suggesting that the association between ethical behaviour and PCC competency may be context‐dependent (Kwame and Petrucka 2021; Bachnick et al. 2018).

Multicollinearity diagnostics conducted on the combined predictor model revealed substantially elevated VIF values across NEBPPR subscales (range: 28.6–125.5), indicating severe multicollinearity when all ethical behaviour dimensions were entered simultaneously into a single model. This finding reflects the conceptual relatedness of the NEBPPR subscales as components of the same underlying ethical construct and is consistent with the possibility of common method variance, given that all variables were collected via self‐report instruments from the same respondents. These diagnostic findings provided the primary methodological rationale for evaluating each NEBPPR subscale in a separate logistic regression model controlling for demographic confounders. The revised models yielded a McFadden pseudo‐*R* ^2^ of 0.419, indicating adequate model fit. The comparatively high explanatory power may partly reflect the homogeneity of the sample and the shared cultural context of Turkish nursing practice, where collectivist values may amplify the alignment between ethical commitment and care delivery. Future studies should examine whether this relationship holds in more resource‐constrained or culturally diverse settings.

The logistic regression models confirmed that ethical behaviour dimensions remained significantly associated with PCC competency after adjusting for confounders such as gender, years of experience, and educational level. This strengthens the internal validity of the findings and indicates that nurses' ethical conduct contributes to PCC outcomes beyond demographic influences. Subscale‐level analysis was theoretically justified, as the conceptual framework (Figure 1) posits that ethical dimensions such as respect for patient autonomy, nonmaleficence, and privacy constitute foundational principles of person‐centred practice (Scholl et al. 2014; Entwistle and Watt 2013). Examining these ethical domains separately provides insight into which principles most strongly influence PCC delivery, enhancing both theoretical depth and practical relevance.

Notably, 78.6% of participants had received ethics education, suggesting that foundational ethics training is well established within Turkish nursing practice. However, participation in certified ethics training remains low (7.1%), reflecting inadequate opportunities for continuing professional development, a gap documented in nursing populations internationally (Ulrich et al. 2010). This gap is concerning, given that nurses who received patient rights education demonstrated significantly higher PCC competency scores (U = 11,436, *p* = 0.001), supporting the broader principle that structured nursing education programs, including those focused on shared decision‐making, can enhance patient‐centred orientations over time (Ulrich et al. 2010; Hsu and Lin 2022). Despite high awareness levels regarding PCC (82.4% reported knowledge, 96.2% considered it necessary), participants identified staff shortages as the primary barrier to implementation (72.5%), consistent with the broader literature identifying staffing adequacy as a primary organizational barrier to PCC implementation (Kwame and Petrucka 2021; Bachnick et al. 2018). The disconnect between nurses' philosophical commitment to PCC and their ability to implement it effectively highlights critical constraints in the healthcare system that require administrative attention. In contrast, some studies have found that ethics training does not consistently translate into improved patient‐centred behaviours, particularly when training is delivered didactically rather than through experiential or reflective methods (Vanlaere and Gastmans 2007). The low rate of certified post‐graduation ethics training in the current sample (7.1%) raises the question of whether the observed association between ethics education and PCC competency is primarily driven by foundational undergraduate training rather than continuing professional development, a distinction with important implications for curriculum design.

The demographic analysis revealed no statistically significant associations between personal or professional characteristics and PCC competency scores in this sample. This contrasts with studies reporting significant associations with some PCC subdimensions (Sançar et al. 2023) and with ICU nurses in Palestine (Bardhia et al. 2025). Several methodological factors may account for this divergence: the narrow experience distribution in the current sample (42.1% with 1–5 years) may have restricted detectable variance; snowball sampling may have yielded a more homogeneous professional profile than probability sampling; and the high prevalence of ethics training (78.6%) may have functioned as an equalizing factor, reducing the explanatory contribution of individual demographics. Consistent with this interpretation (Pakkonen et al. 2023), similarly found no associations between nurses' background characteristics and PCC competency in a Finnish long‐term care sample, a pattern that aligns with McCormack and McCance (2017) person‐centred practice framework, which emphasizes that professional attributes and values, rather than personal demographic characteristics, form the foundational prerequisites for person‐centred practice. However, the absence of statistical significance should not be interpreted as evidence of clinical irrelevance; demographic factors may exert meaningful clinical influence that was not detectable given the sample characteristics. These findings should therefore be interpreted with caution, and future studies employing larger, more diverse, and probability‐based samples would be better positioned to clarify the role of demographic characteristics in PCC competency.

Spearman correlation analyses revealed strong positive associations between all NEBPPR subscales and PCC dimensions. The strongest associations were observed between NEBPPR Total and PCC Total (ρ = 0.683), “benefit‐not‐harm” and PCC Total (ρ = 0.668), and NEBPPR Total and “respecting patients' perspectives” (ρ = 0.644). These findings are consistent with evidence that respect for patient autonomy and protection of patient rights constitute foundational mechanisms linking ethical conduct to person‐centred practice (Scholl et al. 2014; Entwistle and Watt 2013). However, the magnitude of some correlations warrants caution in interpretation. Because both constructs were measured simultaneously using self‐report instruments administered to the same respondents, common method variance may have inflated the observed associations. Future studies should employ multi‐source designs, combining nurse self‐reports with patient ratings or objective behavioural measures, to provide more robust estimates of this relationship.

Beyond the findings of the present study, emerging methodological approaches offer promising avenues for future research on ethical behaviours and patient‐centred care. Advanced data analysis techniques, including natural language processing applied to nurses' narratives, patient feedback, and clinical documentation, may enable more nuanced and ecologically valid assessments of ethical conduct in real‐world nursing practice than self‐report instruments alone can provide (Wang et al. 2024). Integrating such approaches with traditional survey‐based methods could strengthen the measurement of ethical sensitivity and PCC competency while reducing reliance on self‐reported data and the associated risk of common method bias.

These findings carry concrete implications for nursing practice, education, and health policy in Turkiye. At the practice level, the strong associations between ethical behaviour and PCC competency suggest that institutional efforts to reinforce nurses' ethical sensitivity, through structured case discussions, ethics rounds, and reflective practice sessions, may yield measurable improvements in patient‐centred care delivery. At the educational level, the low rate of certified post‐graduation ethics training (7.1%) alongside the significant association between patient rights education and PCC competency points to a critical gap in continuing professional development. Nursing curricula and in‐service training programs should prioritize experiential and reflective ethics education over didactic approaches. At the policy level, the finding that 72.5% of nurses identified staff shortages as the primary barrier to PCC implementation signals that ethical commitment alone is insufficient without adequate institutional support. Healthcare administrators and policymakers in Turkiye should address staffing adequacy as a structural prerequisite for translating nurses' ethical values into consistent person‐centred practice.

### 6.1 Strengths and Limitations

This study has several strengths and limitations. A key strength is the use of validated instruments to assess both ethical behaviour and PCC, thereby enhancing internal validity and enabling theoretically grounded interpretation. The relatively large and diverse sample provides a robust overview of ethical–behavioural influences on PCC competencies in the Turkish nursing context. The analytical revision, replacing the original combined linear regression model with separate logistic regression models for each NEBPPR subscale, represents an additional methodological strength, as it circumvents the severe multicollinearity identified in diagnostic testing (VIF range: 28.6–125.5) and yields more interpretable and valid estimates of each dimension's independent association with PCC competency.

However, several limitations should be acknowledged. The cross‐sectional design prevents causal inference, limiting the findings to associational interpretations. Both instruments are self‐administered and therefore capture nurses' perceived ethical sensitivity and patient‐centred attitudes rather than their objectively observed practices. Accordingly, the high‐scale scores observed in this study may reflect participants' perceived ethical commitment rather than actual behavioural expression in clinical practice. Self‐reported data may have introduced social desirability bias, leading participants to overestimate their ethical and care behaviours. Furthermore, as both constructs were measured simultaneously via self‐report instruments administered to the same respondents, common method bias cannot be excluded as a potential source of variance inflation. The conceptual proximity between ethical behaviour dimensions and PCC competencies also warrants further validation using divergent validity analyses in future instrument development studies. Future studies should employ multi‐source designs that combine nurse self‐reports with patient ratings or objective behavioural assessments to address these concerns.

Although both inpatient and outpatient nurses were eligible for inclusion, the majority of respondents (80.4%) worked in inpatient settings, which may reflect the distribution of the nursing workforce across Turkiye or the clustering patterns inherent to snowball sampling. This compositional imbalance may limit the generalizability of findings to outpatient nursing contexts. Snowball sampling may have led to network homogeneity, limiting the representativeness of the broader nursing population. Additionally, the lack of patient perspectives restricts the interpretation of PCC outcomes, as data were obtained solely from nurses.

### 6.2 Recommendations for Future Research

Future research should incorporate longitudinal and observational designs to capture the temporal and behavioural dynamics between ethical sensitivity and PCC outcomes, moving beyond the associational findings that cross‐sectional designs can establish. The inclusion of patient perspectives and peer assessments would provide a more comprehensive and ecologically valid evaluation of PCC delivery, while reducing reliance on self‐report measures and the associated risk of common method bias.

Given the severe multicollinearity identified among NEBPPR subscales in the present study, future investigations should consider employing latent variable approaches, such as structural equation modelling, that can simultaneously account for the shared variance among ethical behaviour dimensions while estimating their distinct contributions to PCC competency. Hierarchical regression designs may also help disentangle the mediating and moderating effects of ethical climate, institutional culture, and moral distress on PCC performance.

Emerging methodological approaches, including natural language processing applied to clinical narratives and patient feedback data, offer promising avenues for assessing ethical conduct and patient‐centred behaviours with greater ecological validity than self‐report instruments alone (Wang et al. 2024). Mixed‐method approaches could further deepen understanding of contextual influences and ethical decision‐making processes in real‐world nursing practice (Aydogdu 2022).

Finally, expanding continuing education programs and intervention studies focused on ethical leadership and patient rights advocacy, as well as experiential ethics training, may help bridge the gap between ethical awareness and consistent PCC implementation, particularly given the low rate of certified post‐graduation ethics training observed in this sample (7.1%).

## 7 Conclusions

This study provides evidence that nurses' ethical sensitivity in protecting patient rights is significantly associated with PCC competency, and that all five NEBPPR dimensions are independently associated with PCC outcomes in separate logistic regression models. The analytical revisions undertaken, including the identification of severe multicollinearity in the original model (VIF range: 28.6–125.5) and the transition to separate logistic regression models, strengthen the validity and transparency of these conclusions. Although the cross‐sectional design and reliance on self‐report measures preclude causal inference, the associations observed remained robust after controlling for demographic confounders. The findings suggest that ethical commitment and structural support are complementary prerequisites for effective PCC. This has direct implications for nursing education, where ethics training should be prioritized in both undergraduate and continuing professional development with emphasis on experiential approaches. For health policy, staffing adequacy must be recognized as a structural condition for translating ethical values into consistent care delivery. Future research should employ longitudinal designs, incorporate patient perspectives and objective behavioural measures, and utilize advanced analytical approaches to establish causal direction and provide a more complete understanding of the relationship between ethical behaviour and person‐centred care.

## Author Contributions

**Melek Aksut Ozturk:** conceptualization, data curation, writing – original draft, resources, project administration, funding acquisition, visualization, formal analysis, validation, investigation, methodology, software. **Beratiye Oner:** conceptualization, methodology, software, validation, formal analysis, supervision, visualization, project administration, writing – review and editing, data curation.

## Funding

The authors have nothing to report.

## Ethics Statement

The research was conducted with approval from the Lokman Hekim University Non‐Interventional Clinical Research Ethics Committee (Decision Number: 2024/277—Code Number: 2024267). In line with the Declaration of Helsinki, the study's purpose, the protection of personal data and confidentiality, and the voluntary nature of participation were explained to all participants.

## Consent

Informed consent was obtained from all individual participants included in the study.

## Conflicts of Interest

The authors declare no conflicts of interest.

## Supporting information


**Appendix S1:** Demographic and Professional Characteristics of Participants (*n* = 397).
**Appendix S2:** Knowledge and Experience of Participants on Ethics Education, PCC, and Patient Rights (*n* = 397).
**Appendix S3:** Full Logistic Regression Model Example: Attention to Privacy Subscale Predicting Less‐Than‐High PCC Competency, Including Intercept and Demographic Covariates (*n* = 397) Modelled event: less‐than‐high PCC competency (reference category: high PCC competency).

**Data S1:** Supplementary STROBE Statement—Checklist of items that should be included in reports of cross‐sectional studies.

## Data Availability

The data that support the findings of this study are available from the corresponding author upon reasonable request.
