# Supplementary material for: The Relationship Between Nurses' Ethical Behaviours for Protecting Patients' Rights and Patient‐Centred Care Competencies: A Cross‐Sectional Study
Source: Nurs Open. 2026 Aug 13;13(8):e70744. doi: 10.1002/nop2.70744 (PMC13472541; doi:10.1002/nop2.70744)
Supplement: Supplementary file 2 [file NOP2-13-e70744-s002.docx]

**Supplementary Appendix I.** Demographic and Professional Characteristics of Participants (n=397)

| **Variables** | | **n (397)** | **%** |
| --- | --- | --- | --- |
| **Gender** | Female | 269 | 67.8 |
|  | Male | 128 | 32.2 |
| **Age Groups**  Median (IQR): 29 (26–34) | 20-24 | 33 | 8.3 |
|  | 25-29 | 172 | 43.3 |
|  | 30-34 | 101 | 25.4 |
|  | 35-39 | 36 | 9.1 |
|  | 40-58 | 55 | 13.9 |
| **Marital Status** | Married | 227 | 57.2 |
|  | Single | 170 | 42.8 |
| **Education** | Health Vocational High School Graduate | 64 | 16.1 |
|  | Associate degree | 33 | 8.3 |
|  | Bachelor's degree | 237 | 59.7 |
|  | Graduate degree | 63 | 15.9 |
| **Chose Nursing Voluntarily** | Yes | 312 | 78.6 |
|  | No | 85 | 21.4 |
| **Working Region** | Black Sea | 144 | 36.3 |
|  | Ankara | 114 | 28.7 |
|  | Central Anatolia | 52 | 13.1 |
|  | Marmara | 42 | 10.6 |
|  | Aegean | 22 | 5.5 |
|  | Mediterranean | 10 | 2.5 |
|  | Eastern & Southeast Anatolia | 13 | 3.3 |
| **Institution Type** | State hospital | 124 | 31.2 |
|  | City hospital | 101 | 25.4 |
|  | Private (Foundation) hospital | 75 | 19.0 |
|  | Training and research hospital | 48 | 12.1 |
|  | University hospital | 49 | 12.3 |
| **Professional Experience**  Median (IQR): 6 (3–12) | 1-5 years | 167 | 42.1 |
|  | 6-10 years | 116 | 29.2 |
|  | 11-15 years | 54 | 13.6 |
|  | 16-20 years | 28 | 7.1 |
|  | 21-37 years | 32 | 8 |
| **Unit/Department** | Inpatient wards | 137 | 34.5 |
|  | Intensive care units | 120 | 30.2 |
|  | Emergency department | 62 | 15.6 |
|  | Outpatient clinics | 30 | 7.6 |
|  | Specialty units | 26 | 6.6 |
|  | Operating room | 22 | 5.5 |
| **Work Position** | Inpatient clinic nurse | 319 | 80.4 |
|  | Outpatient clinic nurse | 30 | 7.6 |
|  | Specialty nurse | 26 | 6.5 |
|  | Operating room nurse | 22 | 5.5 |
| **Shift Type** | 8-hour shifts | 108 | 27.2 |
|  | 12-16-hour shifts | 53 | 13.3 |
|  | 24-hour shifts | 117 | 29.5 |
|  | Mixed shifts | 119 | 30 |
| **Weekly Working Hours** | <40 hours | 136 | 34.3 |
|  | >40 hours | 261 | 65.7 |
| **Number of Patients per Shift** | 1-5 patients | 178 | 44.8 |
|  | 6-10 patients | 69 | 17.4 |
|  | 11-20 patients | 65 | 16.4 |
|  | 21-40 patients | 36 | 9.1 |
|  | 41-≥81 patients | 49 | 12.3 |

IQR: Interquartile Range; Specialty units: Angio Unit, Delivery Room, Apheresis Unit, Bone Marrow Transplant Unit, Home Health Services

**Supplementary Appendix II.** Knowledge and Experience of Participants on Ethics Education, PCC, and Patient Rights (n=397)

| **Variables** | | **n (397)** | **%** |
| --- | --- | --- | --- |
| **Ethics Training Status** | Yes | 312 | 78.6 |
|  | No | 85 | 21.4 |
| **Location of Ethics Training** | Undergraduate/Graduate Education | 161 | 51.6 |
|  | Current Workplace | 129 | 41.3 |
|  | Certificate Program | 22 | 7.1 |
| **Awareness of PCC Competence** | Yes | 327 | 82.4 |
|  | No | 70 | 17.6 |
| **Perceived Necessity of PCC** | Yes | 382 | 96.2 |
|  | No | 15 | 3.8 |
| **Reasons for Perceived Deficiencies in PCC** | Staff Shortage | 288 | 72.6 |
|  | Equipment Shortage | 37 | 9.3 |
|  | Paperwork | 31 | 7.8 |
|  | Financial Problems | 2 | 0.5 |
|  | Lack of Training | 2 | 0.5 |
|  | Lack of Organizational Support | 2 | 0.5 |
|  | All of the Above | 35 | 8.8 |
| **Patient Rights Education Status** | Yes | 346 | 87.2 |
|  | No | 51 | 12.8 |
| ***Location of Patient Rights Training** | Current Workplace | 197 | 57.8 |
|  | Undergraduate/Graduate Education | 121 | 35.5 |
|  | Certificate Program | 23 | 6.7 |
| **Perceived Sufficiency of Knowledge on Patient Rights** | Yes | 308 | 77.6 |
|  | No | 89 | 22.4 |

PCC: Patient-Centered Care

*Five participants who indicated receiving patient rights education but did not specify the source were excluded from this analysis (n=341).

**Supplementary Appendix III.** Full Logistic Regression Model Example: Attention to Privacy Subscale Predicting Less-Than-High PCC Competency, Including Intercept and Demographic Covariates (n=397) Modeled event: less-than-high PCC competency (reference category: high PCC competency).

| **Variable** | **B** | **SE** | **OR** | **95% CI** | **p** |
| --- | --- | --- | --- | --- | --- |
| Constant | 5.028 | 1.095 | 152.647 | 17.851–1305.301 | <0.001** |
| Gender (Male vs. Female) | 0.256 | 0.347 | 1.292 | 0.655–2.551 | 0.460 |
| Years of Experience | -0.047 | 0.032 | 0.954 | 0.895–1.016 | 0.144 |
| Education Level | -0.209 | 0.185 | 0.811 | 0.564–1.166 | 0.258 |
| Attention to Privacy | -0.368 | 0.062 | 0.692 | 0.613–0.782 | <0.001** |

B: unstandardized logistic regression coefficient; SE: standard error; OR: odds ratio; CI: confidence interval; McFadden Pseudo R² = 0.166. *p<0.05; **p<0.001.
